# Expression of Concern: Custom-made artificial eyes using 3D printing for dogs: A preliminary study

**DOI:** 10.1371/journal.pone.0245080

**Published:** 2021-01-06

**Authors:** The PLOS ONE Editors

After this article [1] was published, concerns were raised about whether the study meets internationally accepted standards of animal research ethics, as is required per *PLOS ONE*’s Editorial Policy. Specifically, questions were raised as to whether the implants and prosthetics evaluated in the study would offer clinical benefits for dogs as compared to conventional eye removal procedures, so as to justify the reported research. Questions were also raised about the scientific and/or clinical justification for using naïve dogs instead of clinical cases to achieve the study’s objectives.

The ethics statement in [1] reports that the animal experiments were approved by the Chungbuk National University Animal Care and Use Committees (Number: CBNUA-1155-18-01) of Laboratory Animal Research Center at Chungbuk National University (Cheongju, Korea). The article did not discuss the above issues which have implications for adherence of the study to *PLOS ONE*’s policies on animal research ethics.

In addition, questions have been raised about aspects of the methods, results, and conclusions:

- Concerns were raised about the adequacy of the analgesic regimen used given the reported observations of post-operative pain. The methods used to assess animal pain were not clearly reported.
- Questions were raised as to whether the conclusions were well-supported in light of the small sample size and the results reported in Table 1.

The *PLOS ONE* Editors are reassessing the article and following up on the above issues in accordance with Committee on Publication Ethics (COPE) guidance. In the meantime, we issue this Expression of Concern.
