# Correction to Expression of Concern: Custom-made artificial eyes using 3D printing for dogs: A preliminary study

**DOI:** 10.1371/journal.pone.0262839

**Published:** 2022-01-13

**Authors:** The PLOS ONE Editors

Since publishing this Expression of Concern [1] on the 2020 research article by Park et al. [2], PLOS has discussed the concerns with the authors and completed an editorial assessment evaluating both scientific and ethics concerns. In this assessment, we obtained input on the issues raised from experts in clinical veterinary research and ethics and welfare standards for animal research.

This Correction resolves several of the issues raised in the Expression of Concern and provides additional information about the remaining unresolved concern.

## Resolved issues

Based on the outcome of our editorial assessment and input received from the experts consulted in this case, the concerns noted in [1] about the scientific justification for the study, the small sample size, and support for the article’s conclusions have been dismissed. Regarding the scientific justification, a consulted expert advised that this was a pilot study to demonstrate proof-of-concept, that the study design was appropriate, and that as the 3D-printed implants were developed for use in animals an assessment of their *in vivo* tolerability was justified from a clinical, ethical, and regulatory perspective. Another expert commented that the use of prostheses is a topic of debate and controversy among veterinary ophthalmologists.

The authors provided the following information about analgesia and the methods used to assess animal pain, thereby resolving the reporting concern noted in the Expression of Concern (“*The methods used to assess animal pain were not clearly reported*”) [1].

- Tramadol was administered at a dose of 4 mg/kg for pre-operative and post-operative analgesia. For post-operative care, tramadol was administered intravenously every 8 hours for the first three days, and thereafter was administered orally every 12 hours for two weeks after surgery. NSAIDs (non-steroidal anti-inflammatory drugs) were not offered due to concerns about gastrointestinal bleeding.
- Animals did not show psychological or behavioral pain symptoms, as assessed using the Colorado State University pain scale [3, 4] and by observing animals for indicators of pain such as body posture and tension, facial expression, appetite, engagement with surroundings, unsettled behavior, or bothering the surgical site.
- Pain was further assessed by observing responses to palpation at the surgical site. These pain assessments were reported in [2] and were categorized using the following criteria:
  - (None): No psychological and behavioral pain was shown. No react when palpate (slightly press) the surgical site for cleaning and disinfecting the eyelid.
  + (Mild): No psychological and behavioral pain was shown. Sometimes react when palpate palpate (slightly press) the surgical site for cleaning and disinfecting the eyelid.
  ++ (Moderate): No psychological and behavioral pain was shown. React when palpate (slightly press) the surgical site for cleaning and disinfecting the eyelid.
  +++ (Severe): Psychological and/or behavioral pain symptoms including abnormal body posture and tension, decreased appetite, non-happy, non-curiosity about surrounding, and etc. were shown.

In the “Dog’s perception” section of the Results [2], a discrepancy was noted between the duration of pain symptoms reported in the text versus Table 1. The authors clarified that the text provides a summary of the results for both dogs whereas Table 1 reports detailed results for each dog. Moderate pain was detected in both dogs until the third day after surgery, as reported in the text. The pain of Dog 1 (EV) improved to mild status from the post-op 4th day, whereas the pain of Dog 2 (EN) improved from the post-op 8th day, in agreement with what was reported in Table 1. The observed pain symptoms for both dogs were resolved within 2 weeks.

## Remaining concern: Analgesia used for *in vivo* studies

Concerns remain about the adequacy of the analgesic regimen used in this study.

An expert whom PLOS consulted advised that many veterinary anesthesiologists and clinicians consider that tramadol as a single agent has poor analgesic effects in dogs. This advisor and the corresponding author both noted that there are published articles that support the use of tramadol, but that its effectiveness in dogs is controversial.

The expert advisors in this case also expressed concern that no rescue analgesia or supplemental analgesia was provided for dogs who continued to exhibit moderate pain for several days after surgery. The authors stated that they did not provide supplemental or rescue analgesia, or reassess the analgesic protocol, during the post-operative period because they did not observe symptoms in the psychological and behavioral pain assessments that required supplemental analgesia according to the Canine Acute Pain Scale [4]. While one of the expert advisors was satisfied with the authors’ comments about pain assessment and analgesia, the other maintained that supplemental analgesia ought to have been provided.

Considering the above information, the *PLOS ONE* Editors remain concerned about the efficacy and adequacy of the analgesic regimen administered in this study. However, we cannot definitively assess based on the information available post hoc whether the level of pain experienced warranted supplemental and/or a different regimen of analgesia, and furthermore this is not an issue on which PLOS can adjudicate. The Expression of Concern [2] stands on the original article [1] to signal to readers that this issue remains unresolved.
