# Supplementary figures and images for: Custom-made artificial eyes using 3D printing for dogs: A preliminary study
Source: PLoS One. 2020 Nov 20;15(11):e0242274. doi: 10.1371/journal.pone.0242274 (PMC7678976; doi:10.1371/journal.pone.0242274)

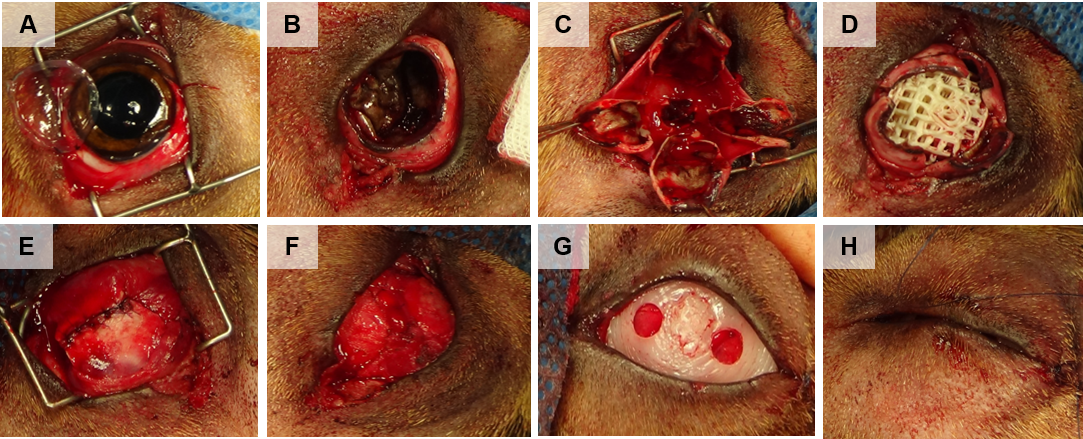

Supplement: S1 Fig — After performing 360° peritomy, sub-Tenon’s blunt dissection and excision of the corneal button were done (A). Ocular contents were scooped out (B) and then four scleral petals were created (C). After inserting the ocular implant (D), the sclera was sutured (E). Two layers of scleral suture were provided for additional cover of the implant. Tenon’s capsule and conjunctiva were sutured respectively (F). The conformer was worn over the conjunctiva (G). Temporal tarsorrhaphy was maintained for 2 weeks (H). (TIF) [file pone.0242274.s001.tif]

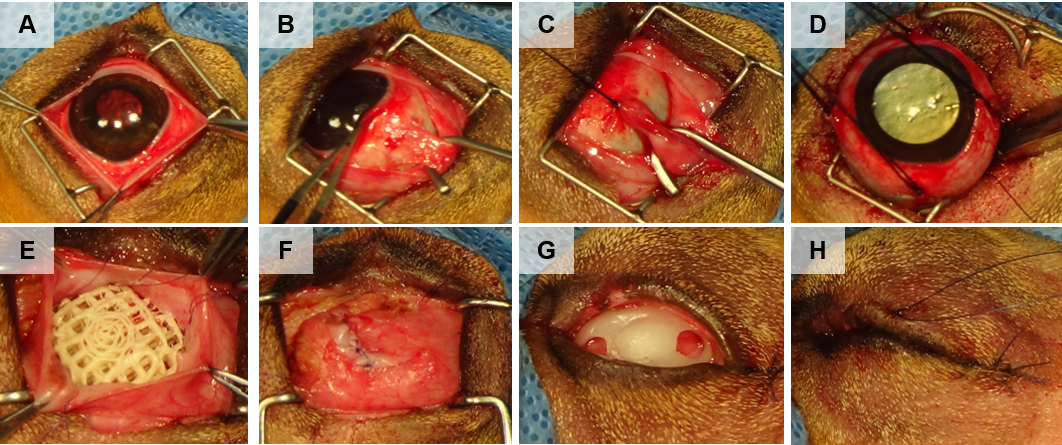

Supplement: S2 Fig — A 360° peritomy and sub-Tenon’s blunt dissection were performed (A). After hooking the four rectus muscles (B), traction suture and stay suture were performed (C). Each of the rectus muscles was transected near its insertion (D). The eyeball was gently lifted using 4 traction sutures, and subsequently, the optic nerve was transected. The ocular implant was inserted (E). Each of the rectus muscles is sutured through the Tenon’s capsule and conjunctiva using double-armed 6–0 polydioxanone. The Tenon’s capsule and conjunctiva were sutured (F). The conformer was worn over the conjunctiva (G). Temporal tarsorrhaphy was maintained for 2 weeks (H). (TIF) [file pone.0242274.s002.tif]
