# Supplementary material for: Custom-made artificial eyes using 3D printing for dogs: A preliminary study
Source: PLoS One. 2020 Nov 20;15(11):e0242274. doi: 10.1371/journal.pone.0242274 (PMC7678976; doi:10.1371/journal.pone.0242274)
Supplement: S1 Table — (DOCX) [file pone.0242274.s003.docx]

**S1 Table. Setting conditions for 3D printing output of orbital implant and ocular prosthesis.**

|  | Implant | Prosthesis |
| --- | --- | --- |
| Nozzle size(mm) | 0.4 | 0.2 |
| Layer height (mm) | 0.2 | 0.1 |
| Output speed (mm/s) | 6 | 10 |
| moving speed (8mm/s) | 8 | 5 |
| Floor layer speed (mm/s) | 3 | 0 |
| Retraction Speed (mm/s) | 1 | 10 |
| minimum moving distance (mm) | 0.5 | 2 |
| Z HOP(mm) | 0.4 | 0.4 |
| Dispenser or extruder temperature (℃) | 100 | 130 |
| Bed temperature (℃) | 10 | Room temperature |
