# Supplementary material for: Custom-made artificial eyes using 3D printing for dogs: A preliminary study
Source: PLoS One. 2020 Nov 20;15(11):e0242274. doi: 10.1371/journal.pone.0242274 (PMC7678976; doi:10.1371/journal.pone.0242274)

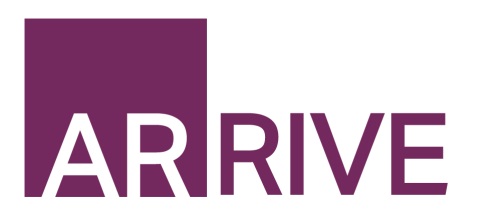


The ARRIVE Guidelines Checklist

Animal Research: Reporting In Vivo Experiments

Carol Kilkenny^1^, William J Browne^2^, Innes C Cuthill^3^, Michael Emerson^4^ and Douglas G Altman^5^

*^1^The National Centre for the Replacement, Refinement and Reduction of Animals in Research, London, UK, ^2^School of Veterinary Science, University of Bristol, Bristol, UK, ^3^School of Biological Sciences, University of Bristol, Bristol, UK, ^4^National Heart and Lung Institute, Imperial College London, UK, ^5^Centre for Statistics in Medicine, University of Oxford, Oxford, UK.*

|  | | ITEM | RECOMMENDATION | Section/ Paragraph |
| --- | --- | --- | --- | --- |
| 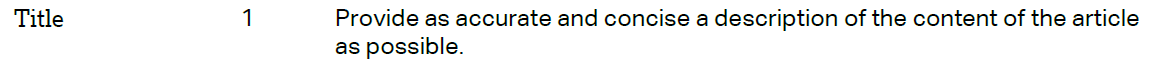 | | | Title |  |
| 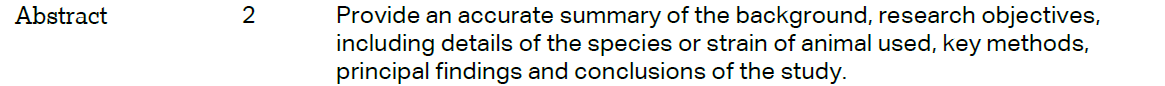 | | | Abstract |  |
| INTRODUCTION | | |  |  |
| 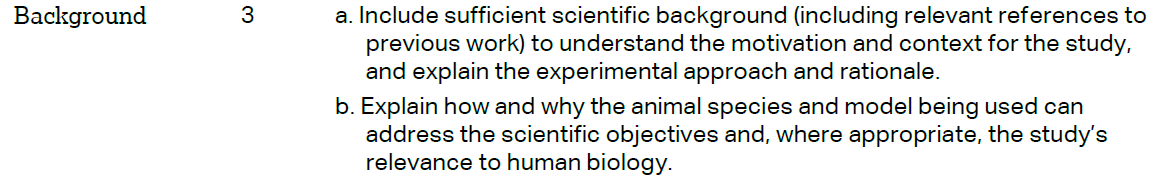 | | | Paragraph1-3  Paragraph1 |  |
| 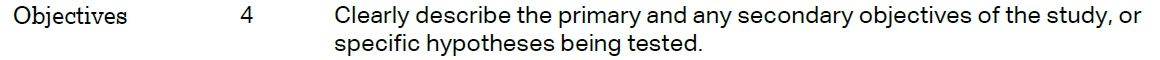 | | | Paragraph3 |  |
| METHODS | | |  |  |
| 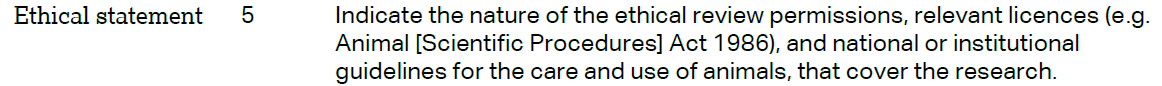 | | | Paragraph 3 |  |
| 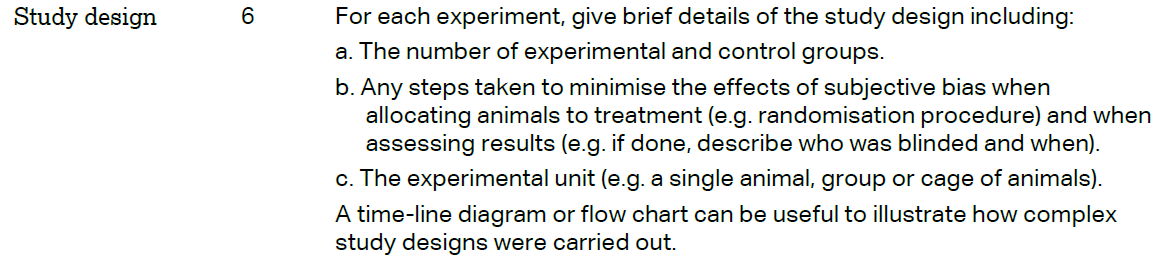 | | | Paragraph3 |  |
| 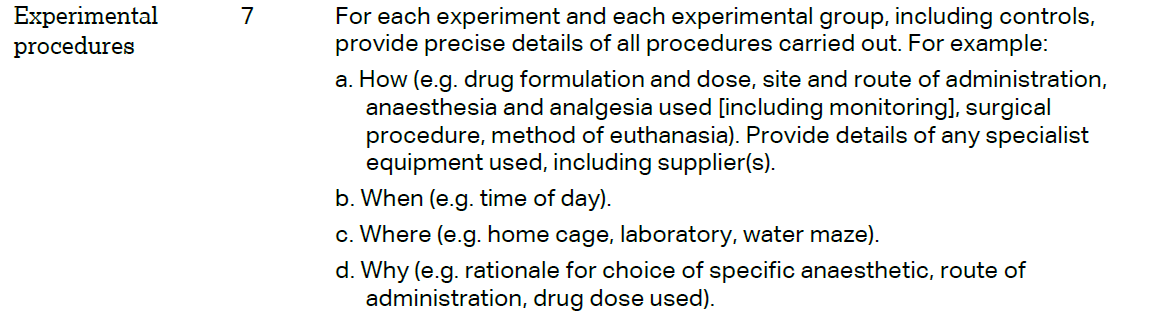 | | | Paragraph3-7 |  |
| 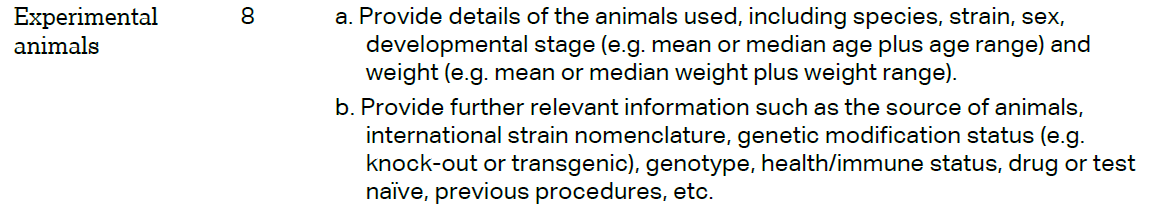 | | | Paragraph3 |  |

The ARRIVE guidelines. Originally published in *PLoS Biology*, June 2010^1^

| 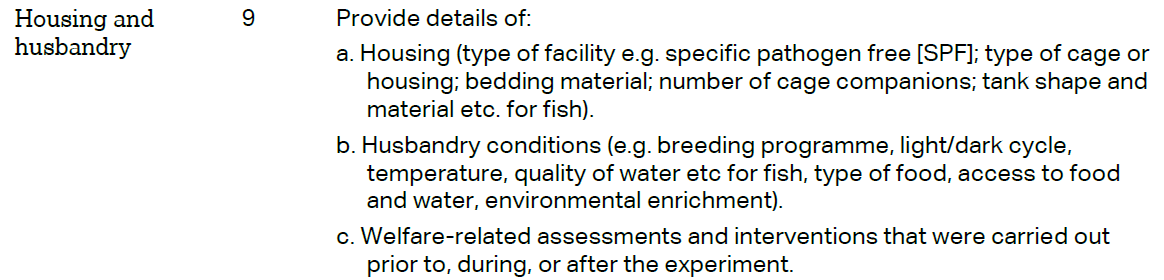 | Paragraph3 | |
| --- | --- | --- |
| 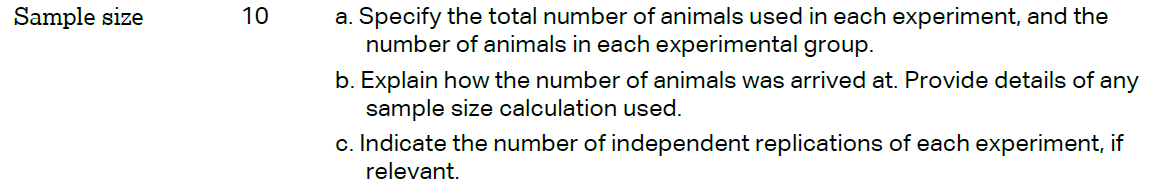 | Paragraph3 | |
| 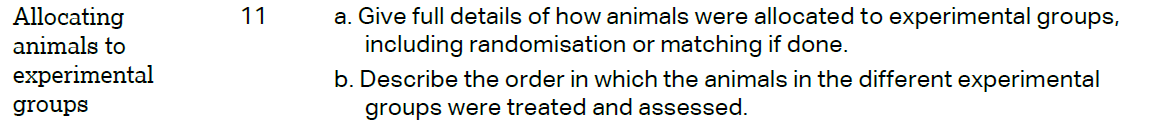 | Paragraph3,5,6 | |
| 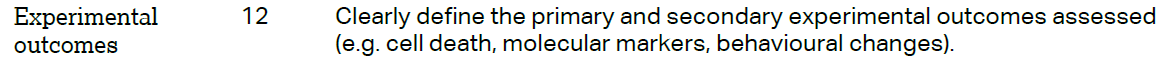 | N/A | |
| 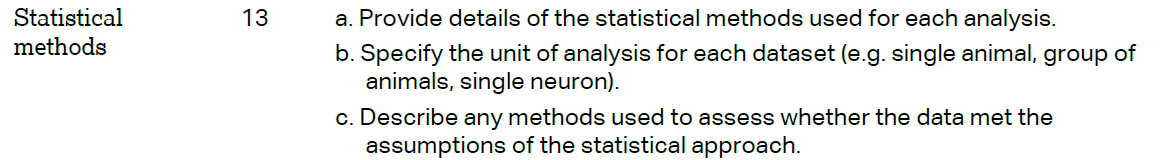 | N/A | |
| RESULTS |  | |
| 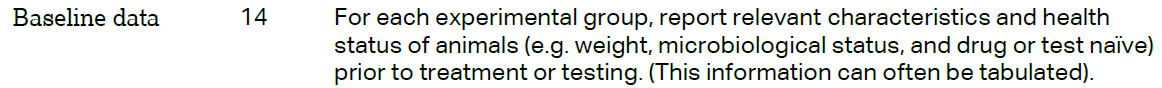 | Paragraph8  (Subhead3, Paragraph3) | |
| 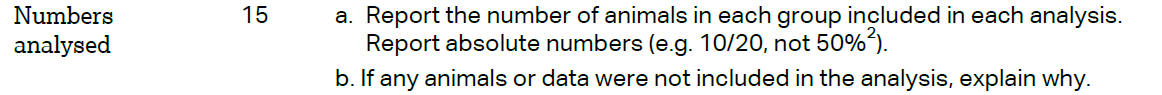 | Paragraph9-11  (Subhead3, Paragraph4-6) | |
| 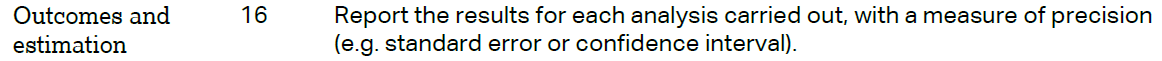 | Paragraph9-11  (Subhead3, Paragraph4-6) | |
| 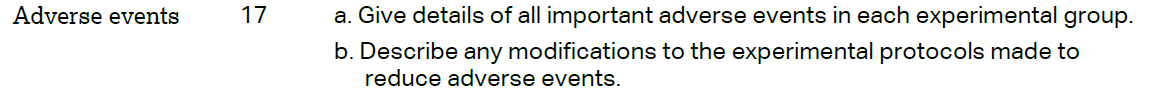 | Paragraph10  (Subhead3, Paragraph5) | |
| DISCUSSION |  | |
| 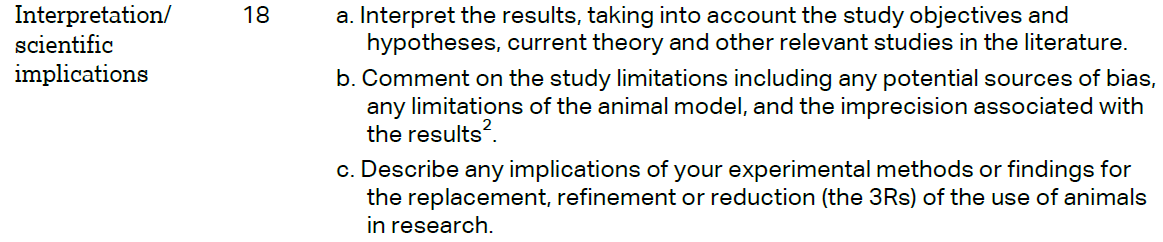 | Paragraph 1-2  Paragraph7-8  N/A | |
| 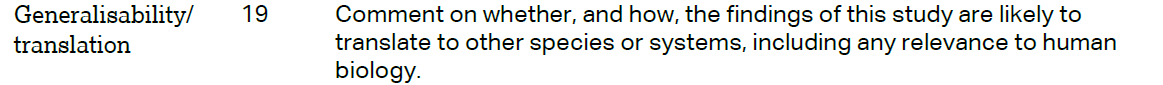 | Paragraph4 | |
| 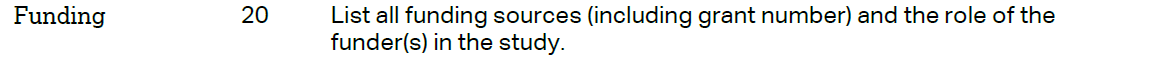 | | Funding |


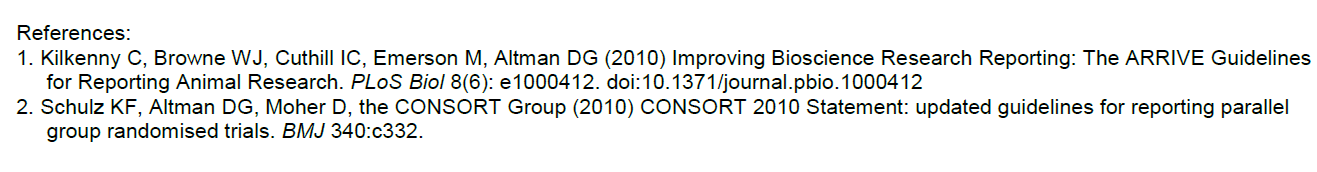

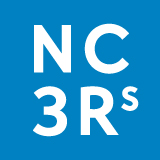

Supplement: S1 File — (DOCX) [file pone.0242274.s004.docx]
