# Supplementary material for: Custom-made artificial eyes using 3D printing for dogs: A preliminary study
Source: PLoS One. 2020 Nov 20;15(11):e0242274. doi: 10.1371/journal.pone.0242274 (PMC7678976; doi:10.1371/journal.pone.0242274)
Supplement: Attachment — Submitted filename: Response to Reviewers.docx [file pone.0242274.s005.docx]

Journal Requirements:

When submitting your revision, we need you to address these additional requirements.

1, Please ensure that your manuscript meets PLOS ONE's style requirements, including those for file naming. The PLOS ONE style templates can be found at

https://journals.plos.org/plosone/s/file?id=wjVg/PLOSOne_formatting_sample_main_body.pdf and

https://journals.plos.org/plosone/s/file?id=ba62/PLOSOne_formatting_sample_title_authors_affiliations.pdf

>> We checked and followed PLOS ONE's style requirements.

2.  Please indicate how often animal care staff monitored the health and well-being of the animals and the criteria used to make such assessments, and provide any additional details regarding housing conditions, feeding and exercise regimens, environmental enrichment if applicable. Thank you for your attention to this request"

>> Line 142-150: We included the required information of animal experiments in our manuscript.

3. We ask that you please consider moving Figures 1 and 2 to Supporting Information due to their graphic nature. Thank you for your consideration

>> We moved Figures 1 and 2 to Supporting Information (S1 and S2 Fig).

Reviewers' comments:

Reviewer's Responses to Questions

5. Review Comments to the Author

Please use the space provided to explain your answers to the questions above. You may also include additional comments for the author, including concerns about dual publication, research ethics, or publication ethics. (Please upload your review as an attachment if it exceeds 20,000 characters)

Reviewer #1: The idea of using 3D printing for making artificial eyes for dogs is a good one; the authors have demonstrated that it works.

Q. People in several professions will be interested in this article. I suggest an amplification of the Materials story in this paper. Why hydroxyapatite ? As discussed by W. Brostow and H.E. Hagg Lobland, Materials: Introduction and Applications, John Wiley & Sons 2017, hydroxyapatite is an important constituents of human and animal bodies; this deserves to be said. Similarly, the authors can explain the reasons for using polycaprolactone vs. PMMA.

>> Thank you for the reviewing our manuscript.

>> In response to the reviewer’s comment, we have improved the material stories in our manuscript.

>> Line 390-397, 400-404, 408-410

Reviewer #2: Dear Editor and authors, thank you for the opportunity offered to me to review this work on the digital workflow in veterinary rehabilitation.
The authors are to be commended for their innovative work, as they sought to integrate the digital protocol into a daily practice of animal eye enucleation treatment.
Nonetheless, as it is, the manuscript presents some major weaknesses that should preclude its publication:

1). I must state that the manuscript was not easy to read and the described technical approach was hard to follow. This fact is surely attributed to the weak structure of this article, as the Materials&Methods and Results sections are a kind of combination.
For instance, you describe the digital data acquisition and go like:
The size of the orbital implant was made to be 75% of the ocular volume by measuring the longest diameter of the eyeball using B-mode ultrasonography, according to a previous report [26]. For size of ocular prosthesis, the X-axis and Z-axis were determined by the diameter.
For me, this belongs surely to the M&M Section
So I suggest that you should clearly state what was done in the M&M section touching upon the following points:
clinical examination
-Surgical approaches and post-op phase
-digital data acquisition
-CAD
-CAM
Then you switch to RESULTS and describe:
-the prosthesis fit
-dogs owns perception
-time and money investments.

>> Thank you for the reviewing our manuscript.

>> In response to the reviewer’s comment, we have revised our M&M and Results sections to describe these points. Please refer to these sections in our manuscript.

>> Line 87, 107, 154, 204, 248, 275-278, 298, 312, 329

2). As for the structure in the Introduction section, I suggest you should change it as well. In the middle of the Introduction section you state the aim of this study, and the start to describe the benefit of 3D printing utilization and its application in human facial prosthetics. I suggest that the aim should be stated clearly and goes to the end of the section.
You go like:
Herein, we aimed to create custom-made artificial eyes including ocular implants and
75 prostheses for dogs using 3D printing in order to overcome these challenges.
I suggest the aim of this clinical trial was not only to create the 3D printed eye prosthese, but also to evaluate their clinical performance in two dog cases and, what was in fact done. Therefore, please rephrase the aim and once again check the introduction section.

>> In response to the reviewer’s comment, we have revised our Introduction. The aim was rephrased and provided at the end of the section.

>> Line 80-82.

3) Currently, custom-made prostheses (CMPs) made of PMMA are mainly used in humans because they are more comfortable and esthetically more acceptable than ready-made prostheses.
Pleasers support this statement with a citation.
>> Line 66-68: In response to the reviewer’s comment, we have added the citation.

4) Currently, 3D printed bio-models are widely used in the medical field because they can provide tactile feedback and reproduce anatomical structures and movements.
You need to provide a strong citation for this statement.

>> Line 76-78: In response to the reviewer’s comment, we have added the citations.

5) The 3D printing technology is used not only for planning surgical procedures, making intraoperative guidance devices, and training, but also for producing facial prostheses of the nose and ear [23-25].
 Citations 23 and 25 seem to be the wrong ones, as they do not relate either to nose or ear prostheses. Please check and consider citing for instance: PMID: 29145528 or PMID: 32611482

>> Line 78-80: In response to the reviewer’s comment, we have corrected these specific citations.

6) 367 Previously, a report scanned the wax model 367 using computed tomography (CT)
I cannot follow this sentence; please rephrase.

>> In response to the reviewer’s comment, we have rephrased the sentence.

>> Line 364-365 : In a previous report, the wax model was produced by computed tomography (CT) scanning, and then the prosthesis was fabricated by 3D printing

7) Please consider describing the other studies in an impersonal way, avoiding such pronouns as he, they. Use the passive voice instead.
>> In response to the reviewer’s comment, we have rephrased our sentences.

>> Line 370-372: To overcome these limitations, a prosthesis was created by scanning the anophthalmic socket using CT, without creating an impression mold in the conventional way.

>> Line 377-378: A digital light processing (DLP) printer with liquid materials was used with high precision and output.

8) Why did you perform both surgical evisceration and enucleation approaches? Was any relevance for this?

>> We have added an explanation for this in the manuscript.

>> Line 424- 430

In this study, we performed two kinds of eye removal surgical methods including evisceration and enucleation because they have been usually done in both human and animal clinics. First, evisceration is performed on unresponsive endophthalmitis or painful eyes with no vision. Evisceration removes the contents of the eye except the sclera, conjunctiva, Tenon’s capsule, extraocular muscles and optic nerve. Second, enucleation removes the entire eyeball except conjunctiva, Tenon’s capsule and extraocular muscles, which is performed mainly in severe trauma, intraocular tumor.

9) Please do a spell check

>> In response to the reviewer's comment, our manuscript has been reviewed by a native English speaker.
