# Supplementary material for: Custom-made artificial eyes using 3D printing for dogs: A preliminary study
Source: PLoS One. 2020 Nov 20;15(11):e0242274. doi: 10.1371/journal.pone.0242274 (PMC7678976; doi:10.1371/journal.pone.0242274)
Supplement: Attachment — Submitted filename: Response to reviewers.docx [file pone.0242274.s006.docx]

Response to reviewers

Reviewer #1:

Q1. I have found an error in English in a sentence "KM Park make the experimental ideas", so please correct.

A> Thank you for the reviewing our manuscript.

We corrected the sentence.

“KM Park designed the experiments.” (Line 464)

Q2. In Ref. 42 the name of the second author should be Hagg Lobland HE, the year 2017.

A> We corrected the author name.

“42. Brostow W, Hagg Lobland HE. Materials: introduction and applications: John Wiley & Sons; 2017.” (Line 507)

Reviewer #2:

Q1. I would like to adress some minor revisions:

Line 330: "No clinical complications such as inflammation, infection, or extrusion of the ocular implant observed during the 6 months after surgery"

The verb "were" is missing

A> Thank you for the reviewing our manuscript.

We corrected the sentence.

“No clinical complications such as inflammation, infection, or extrusion of the ocular implant were observed during the 6 months after surgery (Fig.6A, a).” (Line 330-331)

Q2. Line 69 superior a esthetics. This does not sound good. Please rephrase.

A> We rephrased the sentence.

“Their use in small animals is relatively uncommon although they have shown good results, with few major complications and better appearance.” (Line 69-70)

Q3. Line 354 "We found that using 3D printing, the production time was shortened, the price was lowered, and the labor required was reduced for the production of the artificial eyes" Rephrase the sentence as follows "We found that application of 3D printing allows to decrease the production time...."

A> We rephrased the sentence.

“We found that application of 3D printing allows to decrease the production time, price, and the labor for the production of the artificial eyes” (Line 355-356)

Q4. LIne 364 "In a previous report, wax model was produced by computed tomography (CT) scanning and then fabricated the prosthesis by 3D printing" Rephrase the sentence and omit the word "scanning", as it is not applicable with regards to CT.

A> We rephrased the sentence.

“In a previous report, wax model was produced by computed tomography (CT) and then fabricated the prosthesis by 3D printing” (Line365-366)

Q5. LIne 369 In addition, the soft tissue distortion of the anophthalmic socket could occur owing to the pressure of the molding material during the process of obtaining the impression mold, and an incorrect mold may be obtained. Please omit the last pasrt of the sentence. ("during the process of obtaining the impression mold, and an incorrect mold may be obtained").

A> We rephrased the sentence.

“In addition, the soft tissue distortion of the anophthalmic socket could occur owing to the pressure of the molding material” (Line 369-370)

Q6. Line 376 More recently, prostheses was produced using 3D printing more accurately. Either "Prosthesis was" or "Prostheses were"

A> We corrected the sentence. “prostheses were” (Line 376)

Q7. LIne 442 "the abovementioned" split these two words

A> We corrected the sentence. “the above mentioned” (Line 442)

Q8. line 449. Conclusion must be rephrased. Please avoid using the pronouns like "We" and use the passive voice.

A> We rephrased the sentence.

“In this study, CMP and ocular implants for companion animals were developed using 3D printing technology, overcoming the limitations of a conventional eye removal surgery in dogs.” (Line 449-450)
